# Heterogeneous deniable authenticated encryption for location-based services

**DOI:** 10.1371/journal.pone.0244978

**Published:** 2021-01-06

**Authors:** Chunhua Jin, Ge Kan, Guanhua Chen, Changhui Yu, Ying Jin, Chengjie Xu

**Affiliations:** Faculty of Computer & Software Engineering, Huaiyin Institute of Technology, Huai’an, China; Victoria University, AUSTRALIA

## Abstract

The location-based services can provide users with the requested location information. But users also need to disclose their current location to the location-based service provider. Therefore, how to protect user’s location privacy is a major concern. In this paper, we propose a heterogeneous deniable authenticated encryption scheme called HDAE for location-based services. The proposed scheme permits a sender in a public key infrastructure environment to transmit a message to a receiver in an identity-based environment. Our design utilizes a hybrid encryption method combing the tag-key encapsulation mechanism (tag-KEM) and the data encapsulation mechanism (DEM), which is well adopted for location-based services applications. We give how to design an HDAE scheme utilizing a heterogeneous deniable authenticated tag-KEM (HDATK) and a DEM. We also construct an HDATK scheme and provide security proof in the random oracle model. Comprehensive analysis shows that our scheme is efficient and secure. In addition, we give an application of the HDAE to a location-based services system.

## Introduction

The fast expansion of smart devices and mobile networks makes location-based services (LBSs) an integral part of people’s daily lives. Users utilize LBSs to find points of interests, navigate the destination, and inquire public transportation etc. [1–6]. In all of these requested services, users need to disclose their location information to the location-based service provider (LBSP). Based on location information, LBSP is able to infer some sensitive information about users, such as preferences, social circles, and trajectories. For example, if a user frequently presents location request to the same hospital, the LBSP is able to deduce that the user may have a physical issue.

If the LBSP cooperates with a malicious adversary for pecuniary advantage, there will be significant loss of profits for users. For example, based on the location-based privacy information leaked by a user, a malicious adversary can infer a user’s home address or routine and then commit theft, which seriously threatens user’s personal and property safety. Therefore, protecting users’ location privacy is a major concern.

Authentication plays a very important role in the LBS [7–16]. Only authorized users can access the LBS. Typically, we utilize digital signature technology to achieve authentication. However, there is also non-repudiation in digital signature. That is, the sender cannot deny the message he/she signed. To resolve this issue, deniable authentication [17] is proposed which has two characteristics: (1) the receiver has the capability of identifying whether a given message is from the sender; (2) any third party is incapable of determining whether the given message is from the sender or the receiver even though the third party colludes with the receiver since the receiver is able to generate a probabilistically indistinguishable transcript from the sender. However, in privacy-preserving scenarios, the transmitted message needs to be encrypted to achieve confidentiality. Wu and Li [18] first presented an identity-based DAE scheme to achieve confidentiality as well as deniable authentication in an efficient approach.

### 0.1 Motivation and contribution

In order to make the designed scheme more practical, we require the sender and receiver to be in different cryptographic environments. Concretely, we design a heterogeneous deniable authenticated encryption (HDAE) scheme utilizing tag-KEM and DEM hybrid encryption methods. The proposed scheme permits a sender in a public key infrastructure (PKI) setting to deliver a message to a receiver in an identity-based cryptography (IBC) setting. This construction provides security proof in random oracle model (ROM) under the DBDH and BDH assumptions. Our experimental analysis displays that our scheme has a high efficiency and security. Additionally, we design an LBS scheme utilizing our proposed HDAE scheme. On the one hand, it permits the LBSP to affirm whether the ciphertext of the submitted location request is from the user. On the other hand, any third party cannot determine whether the ciphertext of the submitted location request is from the user or the service provider even though the third party colludes with the LBSP since the LBSP has the capability of generating a probabilistically indistinguishable ciphertext from the user.

### 0.2 Organization

The rest of this paper is arranged below. Section II, Related work is presented. Problem formulation is defined in Section III. We design a formal model for the HDAE in Section IV. Section V, a security model for the HDATK is depicted. An HDAE design is presented in Section VI, and we design an HDATK scheme in Section VII. Performance analysis is discussed in Section VIII. Section IX, we give an HDAE application to the LBS. Conclusion is drawn in Section X.

## 1 Related work

Related notions, hybrid encryption, deniable authenticated encryption, and heterogeneous deniable authentication are introduced.

Hybrid encryption constitutes a key encapsulation mechanism (KEM) and a data encapsulation mechanism (DEM). The KEM encrypts a session key by a public key, whereas the DEM encrypts the real data by a session key. For large messages, hybrid encryption is the best choice. Cramer and Shoup [19] designed practical and provably secure hybrid KEM/DEM schemes. Abe et al. [20] put forward to a more efficient tag-KEM/DEM scheme. Then, many KEM/DEM schemes [21–28] have been proposed. These designs support both components modular design. Sahai et al. [29] put forward to a tag-KEM/DEM scheme by a non-interactive proof method. The proposed scheme can encrypt message with arbitrary length. Baek et al. [30] presented a stateful KEM-DEM scheme. It is highly effective by utilizing a state to produce the random parameters.

Deniable authentication encryption (DAE) is a cryptographic primitive which can accomplish concurrently public key encryption and deniable authentication. Its cost is lower than that needed by deniable authentication-then-encryption manner. The DAE can achieve deniable authentication and confidentiality simultaneously which is well adopted for privacy-protecting scenarios.

Li et al. [31] constructed a DAE scheme with formal security proof. They also constructed an email system based on the designed DAE scheme. Jin et al. [32] constructed a DAE scheme which can realize simultaneously deniable authentication, confidentiality, and ciphertext anonimity. Rasmussen and Gasti [33] proposed a DAE based on two encryption schemes with strong and weak properties. Recently, Huang et al. [34] constructed a DAE scheme for privacy protection with formal security proof. The above mentioned schemes are all in the PKI environment which has public key management problems, including distribution, storage, and revocation. To resolve this issue, a number of identity-based deniable authenticated encryption (IBDAE) schemes have been constructed. Wu and Li [18] constructed an IBDAE scheme which provided formal security proof. Li et al. [35] (denoted by LZJ) proposed an IBDAE scheme for e-mail system. In their scheme, they utilize tag-KEM/DEM hybrid encryption technology which is more suitable for actual applications. Jin and Zhao [36] designed an IBDAE scheme which admitted formal security proof. The aforementioned schemes have key escrow problems, i.e., a third party called private key generator (PKG) knows all user’s private key. To avoid this problem, a certificateless deniable authenticated encryption (CLDAE) scheme [37] has been designed. Recently, Chen et al. [38] proposed a certificateless hybrid KEM/DEM scheme. It separates two parts to provide better security and efficiency.

The aforementioned DAE schemes have a common feature, i.e., the entities of these schemes are all in the same cryptosystem. Such characteristic makes these schemes not well suitable for the LBS system. Li et al. [39] (denoted by LHO) designed two heterogeneous deniable authentication (HDA) schemes. Their designed schemes allowed batch verification to accelerate the authenticators’ verification. Jin et al. [40] constructed an HDA scheme. In their scheme, a sender in a CLC setting delivered a message to a receiver in an IBC setting. However, these schemes do not achieve confidentiality.

## 2 Problem formulation

### 2.1 System and security models

**Fig 1:**
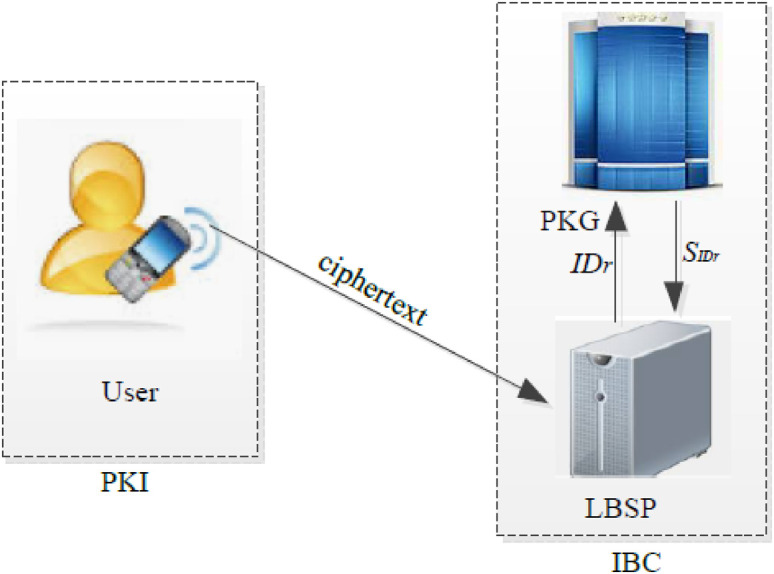
System model.

There are three entities in the HDAE as shown in Fig 1: a user, an LBSP, and a trusted third party PKG. The location information and the corresponding ciphertext are produced by the user, and the ciphertext are sent to the LBSP. The LBSP can identify the received ciphertext is from the user and generate a probabilistically indistinguishable ciphertext from the user. The PKG is mainly responsible for generating system parameters and LBSP’s private key.

To obtain the location-based service that supports privacy-preserving, in the proposed system model, the user sends the ciphertext of location-requested information to the LBSP. Then the LBSP decrypts the received ciphertext and checks whether the decrypted message is location-requested information or a failure symbol ⊥.

### 2.2 Threat model and security goals

We define an adversary which will act as a user to learn the requested location information of other users. The LBSP is honest-but-curious. It means that it follows the designed scheme, but it may collude with a third party for economic benefits. Additionally, the collusion attack between the LBSP and a third party is concerned in the proposed security goals. Specially, two kinds of security requirements are considered in the constructed scheme.

- Confidentiality: Any information about the submitted location information of a ciphertext cannot be learned by any third party other than the involved entities;
- Deniable authentication: The LBSP has a capability of determining a ciphertext is from the user and creating a ciphertext that is probabilistically indistinguishable from the user.

## 3 PI-HDAE

We describe security notions for the HDAE in this section. In the designed HDAE scheme, a sender in a PKI environment, while a receiver in an IBC environment. PI-HDAE is denoted by this kind of DAE as follows.

### 3.1 Syntax

A PI-HDAE scheme comprises five algorithms below:

*Setup*: Given system parameter 1^*k*^, the PKG obtains the *params* and a master private key *s*. In other algorithms, we neglect *params* due to they are public.

*PKI-KG*: A user belongs to the PKI setting elects a secret key *sk* and calculates its public key *pk*.

*IBC-KE*: A user in the IBC setting transmits its identity *ID* to the PKG who computes its private key *S*_*ID*_ and securely passes it to the user. Here, let the user’s public key be its identity *ID*.

*Deniable-Authenticated-Encrypt(DAE)*: Given a message *m*, a sender’s secret key *sk*_*s*_, public key *pk*_*s*_, and a receiver’s identity *ID*_*r*_, the sender obtains a ciphertext *σ*.

*Deniable-Authenticated-Decrypt(DAD)*: Given a ciphertext *σ*, a sender’s public key *pk*_*s*_, a receiver’s identity *ID*_*r*_, and its private key $S_{ID_{r}}$, the receiver obtains a message *m* or a symbol ⊥.

If *σ* = *DAE*(*m*, *sk*_*s*_, *pk*_*s*_, *ID*_*r*_), then *m* = *DAD*(*σ*, *pk*_*s*_, *ID*_*r*_,$S_{ID_{r}}$).

### 3.2 Security notions

We rewrite the notions [35] to meet our scheme. For confidentiality, the standard security concept, indistinguishability against adaptive chosen ciphertext attacks (IND-CCA2) is employed in our construction.

For IND-CCA2 security in a PI-HDAE scheme, it is assumed that this game below is between an adversary F with its challenger C.

“IND-CCA2” game (Game-I):

**Setup**. C performs *Setup* algorithm to get *params*, releases it to F and saves *s*. C also executes the PKI-KG algorithm to obtain a sender’s private/public key pair ($sk_{s}^{*}$, $pk_{s}^{*}$). Then it passes $pk_{s}^{*}$ to F.

**Phase 1**. F adaptively issues the queries below.

- Key extraction queries: F picks an identity *ID*. C obtains the private key *S*_*ID*_ by running an IBC-KE algorithm and transmits it to F.
- DAE queries: F selects a receiver’s identity *ID*_*r*_, and a message *m*. Then C executes DAE(*m*, $sk_{s}^{*}$, $pk_{s}^{*}$, *ID*_*r*_) and transmits the result *σ* to F.
- DAD queries: F selects a ciphertext *σ*, and a receiver’s identity *ID*_*r*_. C obtains $S_{ID_{r}}$ by implementing key extraction algorithm. It then transmits *σ* = DAD(*σ*, $pk_{s}^{*}$, *ID*_*r*_, $S_{ID_{r}}$) to F (the resulting ⊥ indicates *σ* is invalid).

**Challenge**. F determines when Phase 1 ends. F creates a challenge identity $ID_{r}^{*}$ and two messages (*m*_0_, *m*_1_). In phase 1, it does not support to request a key extraction query on $ID_{r}^{*}$. C randomly picks *b* ∈ {0, 1}, computes σ* = DAE(*m*_*b*_, $sk_{s}^{*}$, $pk_{s}^{*}$, *ID*_*r*_) and outputs σ* to F.

**Phase 2**. F makes queries as in Phase 1 except it neither requests a key extraction query on identity $ID_{r}^{*}$ nor executes a DAD query on (σ*, $pk_{s}^{*}$, $ID_{r}^{*}$).

**Guess**. F returns *b*′, and it wins the game if *b*′ = *b*.

F’s advantage is

$$Adv_{PI-HDAE}^{IND-CCA2}\left(F\right)=|2Pr[b^{\prime }=b]-1|,$$

where *Pr*[*b*′ = *b*] expresses the probability.

**Definition 1**. A PI-HDAE scheme is IND-CCA2 secure if there is a probabilistic polynomial time (PPT) adversary F wins “IND-CCA2” game with a negligible advantage.

In the aforementioned definition, F is permitted to gain the sender’s private key $S_{ID_{s}}$ [41]. Namely, the confidentiality is retained if the $S_{ID_{s}}$ is compromised.

For deniable authentication, the security concept, deniable authentication against adaptive chosen message attacks (DA-CMA) is employed in our construction.

For DA-CMA in a PI-HDAE scheme, this game below is between F and C.

“DA-CMA” game (Game-II):

**Setup**. This is identical to Game-I.

**Attack**. This is identical to Game-I.

**Forgery**. F creates a pair (σ*,$ID_{r}^{*}$). F succeeds if the conditions below are satisfied:

1. DAD(σ*,$pk_{s}^{*}$,$ID_{r}^{*}$,$S_{ID_{r}}$) = m*.
2. F has not issued a key extraction query on $ID_{r}^{*}$.
3. F has not issued a DAE query on (m*, $ID_{r}^{*}$).

F’s advantage is defined as the probability that it will win.

**Definition 2**. A PI-HDAE scheme is DA-CMA secure if there is a PPT adversary F wins the “DA-CMA” game with a negligible advantage.

In the aforementioned definition, F does not issue a key extraction query on the identity $ID_{r}^{*}$. This is for deniability. In other words, the two parties involved communication are able to produce a transcript with indistinguishable probability.

### 3.3 Data Encapsulation Mechanism (DEM)

Two algorithms are included in a DEM.

- Enc: Given 1^*k*^, a message *m*, and a key *K*, this algorithm outputs a ciphertext *c*. It is denoted as *c* = *Enc*(*K*, *m*).
- Dec: Given a key *K*, and a ciphertext *c*, this algorithm outputs a message *m* or ⊥.

For a DEM, the security concept, indistinguishability against passive attackers (IND-PA) is employed in our construction. The game below is between A and C.

IND-PA game (Game-III):

**Setup**. A transmits two messages (*m*_0_, *m*_1_).

**Challenge**. C picks *K*, *β* ∈ {0, 1}, and outputs a challenge ciphertext c* = *Enc*(*K*, *m*_*β*_) to A.

**Guess**. A returns *β*′, and it will win the game if *β*′ = *β*.

A’s advantage is

$$Adv_{DEM}^{IND-PA}\left(A\right)=|2Pr[\beta^{\prime }=\beta ]-1|,$$

where *Pr*[*β*′ = *β*] expresses the probability.

**Definition 3**. A DEM is DA-CPA secure if there is a PPT adversary A wins “DA-CPA” game with a negligible advantage.

## 4 PI-HDATK

The security notions for heterogeneous deniable authenticated tag-KEM (HDATK) are given in this section. In the designed HDATK scheme, a sender belongs to a PKI setting, while a receiver belongs to an IBC setting. PI-HDATK is denoted by this kind of DATK scheme as follows.

### 4.1 Syntax

A PI-HDATK scheme comprises six algorithms below:

*Setup*: Given 1^*k*^, the PKG obtains the *params* and a master private key *s*. Due to *params* are public, we neglect them in other algorithms.

*PKI-KG*: A user in the PKI setting calculates a secret/public key pair (*sk*, *pk*).

*IBC-KE*: A user in the IBC setting transmits its identity *ID* to the PKG who computes its private key *S*_*ID*_ and securely transmits it to the user. Here, we assume that the user’s public key is its identity *ID*.

*Sym*: Given a sender’s secret key *sk*_*s*_, public key *pk*_*s*_, and a receiver’s identity *ID*_*r*_, the sender produces an encryption key *K* and state information *ω*.

*Encap*: Given a tag *τ* and the state information *ω*, the sender creates an encapsulation *ϕ*.

*Decap*: Given a sender’s public key *pk*_*s*_, a receiver’s identity *ID*_*r*_, private key $S_{ID_{r}}$, a tag *τ*, and an encapsulation *ϕ*, the receiver outputs *K* or ⊥.

If (*k*, *ω*) = *Sym*(*sk*_*s*_, *pk*_*s*_, *ID*_*r*_) and *ϕ* = *Encap*(*ω*, *τ*), then $K=Decap(\phi ,\tau ,pk_{s},ID_{r},S_{ID_{r}})$.

### 4.2 Security notions

The confidentiality and deniable authentication should be satisfied for the PI-HDATK scheme. For IND-CCA2 security in a PI-HDATK scheme, it is assumed that this game below is between F and C.

“IND-CCA2” game (Game-IV):

**Setup**. C performs *Setup* algorithm, delivers *params* to F and saves *s*. C also executes PKI-KG algorithm to obtain a sender’s private/public key pair ($sk_{s}^{*}$, $pk_{s}^{*}$). Then it delivers $pk_{s}^{*}$ to F.

**Phase 1**. F adaptively issues queries below.

- Key extraction queries: This is identical to Game-I.
- Symmetric key generation queries: F submits a receiver’s identity *ID*_*r*_ to C. C then performs $\left(K,\omega \right)=Sym\left(sk_{s}^{*},pk_{s}^{*},ID_{r}\right)$, stores the state information *ω*, and sends the key *K* to F.
- Encapsulation queries: F picks a tag *τ*. If *ω* is not matched, C outputs ⊥. If matched, C deletes the exist one and produces *ϕ* = *Encap*(*ω*, *τ*)
- Decapsulation queries: F picks an encapsulation *ϕ*, a receiver’s identity *ID*_*r*_, and a tag *τ*. C produces $S_{ID_{r}}$ by performing key extraction algorithm. It outputs the result of Decap(*ϕ*, *τ*, $pk_{s}^{*}$, *ID*_*r*_, $S_{ID_{r}}$) to F.

**Challenge**. F determines when Phase 1 is over. F then outputs a challenge identity $ID_{r}^{*}$. In phase 1, it does not support to request a key extraction query on $ID_{r}^{*}$. C executes (*K*_1_, ω*) = Sym($sk_{s}^{*}$,$pk_{s}^{*}$,$ID_{r}^{*}$), picks *b* ∈ {0, 1}, $K_{0}\in K_{PI-HDATK}$, and passes *K*_*b*_ to F. when F obtains *K*_*b*_, it will issue the identical queries as before. F then returns a tag τ*. C calculates a challenge encapsulation ϕ* = Encap(ω*, τ*) and outputs it to F.

**Phase 2**. F makes queries as in Phase 1 except it neither requests a key extraction query on identity $ID_{r}^{*}$ nor executes a decapsulation query on (ϕ*, τ*, $pk_{s}^{*}$, $ID_{r}^{*}$).

**Guess**. F returns *b*′, and it wins the game if *b*′ = *b*.

F’s advantage is

$$Adv_{PI-HDATK}^{IND-CCA2}\left(F\right)=|2Pr[b^{\prime }=b]-1|,$$

where *Pr*[*b*′ = *b*] expresses the probability.

**Definition 4**. A PI-HDATK scheme is IND-CCA2 secure if a PPT adversary F wins “IND-CCA2” game with negligible advantage.

In the above definition, it is allowed that F gets the sender’s secret key $S_{ID_{s}}$. Namely, the confidentiality is maintained if $S_{ID_{s}}$ is compromised.

For deniable authentication, the security concept, deniable authentication against adaptive chosen message attacks (DA-CMA) is employed in our design.

For DA-CMA security in a PI-HDATK scheme, it is assumed that this game below is played between F with C.

“DA-CMA” game(Game-V):

**Setup**. This is identical to Game-III.

**Attack**. This is identical to Game-III.

**Forgery**. F creates an element (ϕ*, τ*, $ID_{r}^{*}$). F succeeds if the contexts below are met:

1. DAD(σ*,$pk_{s}^{*}$,$ID_{r}^{*}$) = m*.
2. F has not issued a key extraction query on $ID_{r}^{*}$.
3. F has not issued a DAE query on (m*, $ID_{r}^{*}$).

F’s advantage is defined as the probability that it will win.

**Definition 5**. A PI-HDATK scheme is DA-CMA secure if a PPT adversary F wins the “DA-CMA” game with a negligible advantage.

In the aforementioned definition, F does not issue a key extraction query on $ID_{r}^{*}$. This is for deniability. That is, the two parties involved communication are able to produce an indistinguishable transcript.

## 5 A hybrid PI-HDAE scheme

**Fig 2:**
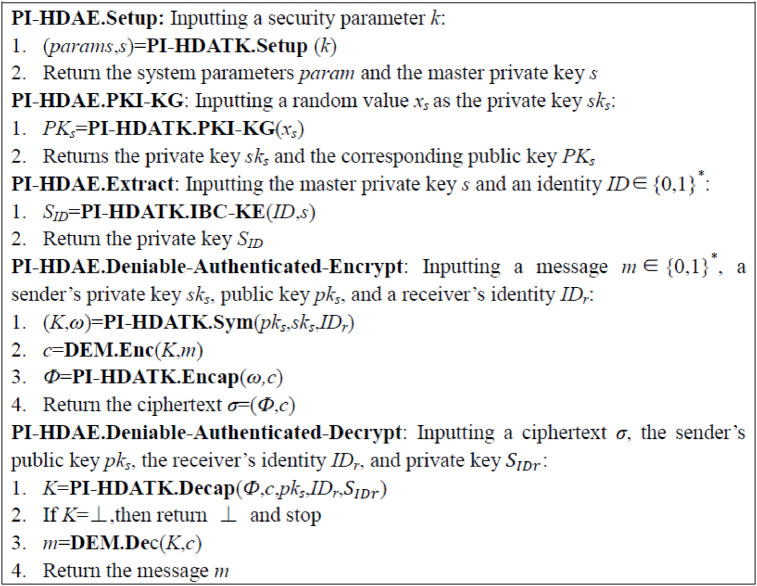
Construction of PI-HDAE from PI-HDATK and DEM.

Fig 2 depicts a hybrid PI-HDAE scheme that constitutes a PI-HDATK and a DEM. In DEM part, the ciphertext is a tag. This construction provides simple description. Theorems 1 and 2 present the security consequences.

**Theorem 1**. Let a hybrid PI-HDAE scheme constitute a PI-HDATK and a DEM which are IND-CCA2 and IND-CPA secure, respectively, PI-HDAE is IND-CCA2 secure. to be specific, we receive

$$Adv_{PI-HDAE}^{IND-CCA2}\left(F\right)=Adv_{PI-HDATK}^{IND-CCA2}\left(C_{1}\right)+Adv_{DEM}^{IND-PA}\left(C_{2}\right),$$

*Proof*: See Appendix 1.

**Theorem 2**. Let a PI-HDAE constitutes a PI-HDATK and a DEM. If PI-HDATK is DA-CMA secure, PI-HDAE is also DA-CMA secure. to be specific, we receive

$$Adv_{PI-HDAE}^{DA-CMA}\left(F\right)\leq Adv_{PI-HDATK}^{DA-CMA}\left(C\right),$$

*Proof*: Refer to Appendix 2.

## 6 A PI-HDATK scheme

**Fig 3:**
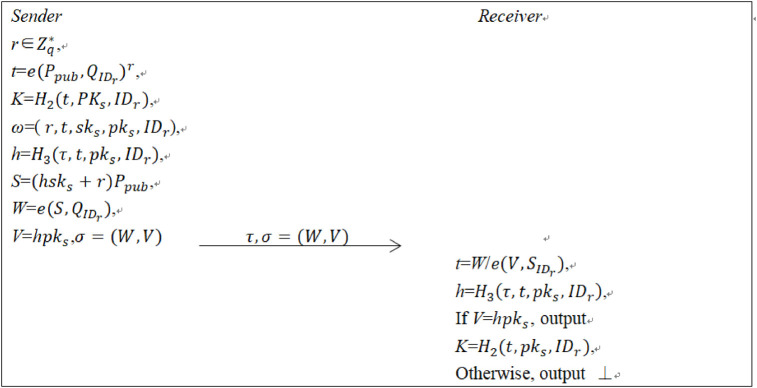
The main contribution of PI-HDATK.

There are six algorithms to describe our proposed scheme. Fig 3 shows the main description. In DEM part, a tag is the ciphertext. This construction provides simple description and realizes better universal security.

### 6.1 Basic knowledge

In this section, we provide bilinear pairings properties, decisonal bilinear Diffie-Hellman problem (DBDHP), and bilinear Diffie-Hellman problem (BDHP).

Let *G*_1_, *G*_2_ be an additive group and a multiplicative group, respectively. *P* is a generator of *G*_1_, and *G*_1_ as well as *G*_2_ have the same prime order *q*. A bilinear pairing is a map *e*: *G*_1_ × *G*_1_ → *G*_2_ with the following properties:

1. Bilinearity: *e*(*aP*, *bQ*) = *e*(*P*, *Q*)^*ab*^ for all $P,Q\in G_{1},a,b\in Z_{q}^{*}$.
2. Non-degeneracy: There exists *P*, *Q* ∈ *G*_1_ such that *e*(*P*, *Q*) ≠ 1.
3. Computability: There is an efficient algorithm to compute *e*(*P*, *Q*) for all *P*, *Q* ∈ *G*_1_

The modified Weil and Tate pairings are the admissible maps ([42–48] offer more information). This scheme’s security depends on the difficulty of dealing with the flllowing problems.

**Definition 1. Decisional Bilinear Diffie-Hellman Problem (DBDHP)**. In the light of bilinear pairings basic definition as above mentioned, DBDHP is to determine *θ* = *e*(*P*, *P*)^*abc*^ given (*P*, *aP*, *bP*, *cP*) with $a,b,c,\theta \in Z_{q}^{*}$.

**Definition 2. Bilinear Diffie-Hellman Problem (BDHP)**. In the light of bilinear pairings basic definition as above mentioned, BDHP is to calculate *e*(*P*, *P*)^*abc*^ given (*P*, *aP*, *bP*, *cP*) with $a,b,c\in Z_{q}^{*}$.

### 6.2 Our scheme

**Setup**. Given *G*_1_, *G*_2_, *P*, and *e* as in Subsection A of Section VII. Let *k* be a security parameter (*q* ≥ 2^*k*^) and *n* be a a DEM’s key length. *H*_1_, *H*_2_, *H*_3_ are three cryptographic hash functions, where *H*_1_: {0, 1}* → *G*_1_, *H*_2_: {0, 1}* × *G*_1_ × *G*_2_ → {0, 1}^*n*^ and $H_{3}:{\left\{0,1\right\}}^{*}\times G_{1}\times G_{2}\to Z_{q}^{*}$. The KGC randomly selects a master key $s\in Z_{q}^{*}$ and calculates *P*_*pub*_ = *sP*. The public *params* are (*G*_1_, *G*_2_, *e*, *q*, *n*, *k*, *P*, *P*_*pub*_, *H*_1_, *H*_2_, *H*_3_) and a master private key is *s*.

**PKI-KG**. A user belongs to a PKI setting elecets $x_{i}\in Z_{q}^{*}$ randomly as its secret key *sk*_*i*_, and calculates *pk*_*i*_ = *sk*_*i*_ *P* as its public key. Here, *i* = *s* denotes the sender, and *pk*_*s*_ = *x*_*s*_ *P*, *sk*_*s*_ = *x*_*s*_ denotes the sender’s public/private key pair.

**IBC-KE**. A user belongs to an IBC setting gives its identity *ID* to the PKG. The PKG calculates its private key *SK*_*ID*_ = *sQ*_*ID*_(*Q*_*ID*_ = *H*_1_(*ID*)) and securely transmits it to the user. Here, *ID*_*r*_ denotes the receiver, and *pk*_*r*_ = *ID*_*r*_ $sk_{r}=S_{ID_{r}}$ denote the receiver’s public and private key.

**Sym**. Given a sender’s private/public key pair (*sk*_*s*_, *pk*_*s*_), and a receiver’s identity *ID*_*r*_, the algorithm below is done.

1. Pick $r\in Z_{q}^{*}$.
2. Compute $t=e{\left(P_{pub},Q_{ID_{r}}\right)}^{r}$.
3. Calculate *K* = *H*_2_(*t*, *pk*_*s*_, *ID*_*r*_).
4. Return *K* and *ω* = (*r*, *t*, *sk*_*s*_, *pk*_*s*_, *ID*_*r*_).

**Encap**. Given a tag *τ* and the state information *ω*, the algorithm below is done.

1. Compute *h* = *H*_3_(*τ*, *t*, *pk*_*s*_, *ID*_*r*_).
2. Compute *S* = (*hsk*_*s*_ + *r*)*P*_*pub*_.
3. Compute $W=e(S,Q_{ID_{r}})$.
4. Compute *V* = *hpk*_*s*_.
5. Compute *σ* = (*W*, *V*).

**Decap**. Given a tag *τ*, an encapsulation *σ*, a sender’s public key *pk*_*s*_, a receiver’s private key $S_{ID_{r}}$, identity *ID*_*r*_, the algorithm below is executed.

1. Compute $t=W/e(V,S_{ID_{r}})$.
2. Compute *h* = *H*_3_(*τ*, *t*, *pk*_*s*_, *ID*_*r*_).
3. If *V* = *hpk*_*s*_, output *K* = *H*_2_(*t*, *pk*_*s*_, *ID*_*r*_); if not, return the symbol ⊥.

The consistency of the designed HDATK scheme can be verified. Because $W=e(S,Q_{ID_{r}})$, *V* = *hpk*_*s*_, we can get

$$\begin{array}{ccc} t & = & W/e\left(V,S_{ID_{r}}\right)=e\left(S,Q_{ID_{r}}\right)/e\left(hpk_{s},S_{ID_{r}}\right)\\ & = & e\left(\left(hx_{s}+r\right)P_{pub},Q_{ID_{r}}\right)/e\left(hpk_{s},sQ_{ID_{r}}\right)\\ & = & e\left(hx_{s}P_{pub},Q_{ID_{r}}\right)e\left(rP_{pub},Q_{ID_{r}}\right)/e\left(hx_{s}sP,Q_{ID_{r}}\right)\\ & = & e\left(hx_{s}sP,Q_{ID_{r}}\right)e\left(rP_{pub},Q_{ID_{r}}\right)/e\left(hx_{s}sP,Q_{ID_{r}}\right)\\ & = & e{\left(P_{pub},Q_{ID_{r}}\right)}^{r} \end{array}$$

### 6.3 Security

Theorems 3 and 4 offer the security consequences for PI-HDATK.

**Theorem 3**. Under DBDH assumption, in ROM, F wins the IND-CCA2 game with a non-negligible advantage *ϵ*_*datk*_ when issuing $q_{H_{i}}$ queries to *H*_*i*_ (*i* = 1, 2, 3), *q*_*ke*_ key extraction queries, *q*_*gsk*_ generation symmetric key queries, *q*_*ke*_ key encapsulation queries, and *q*_*kd*_ key decapsulation queries in a time *t*, C resolves DBDH problem with probability

$$\epsilon_{datk}\geq \frac{\epsilon -q_{kd}/2^{k-1}}{2q_{H_{1}}}$$

within *t*′ ≤ *t* + *O*(*q*_*gsk*_ + *q*_*ke*_ + *q*_*kd*_)*t*_*p*_, in which *t*_*p*_ is one paring computation.

*Proof*: Refer to Appendix 3.

**Theorem 4**. Under BDH assumption, in ROM, F has a non-negligible advantage $\epsilon_{datk}\geq 10\left(q_{ke}+1\right)\left(q_{ke}+q_{H_{3}}\right)q_{H_{1}}/\left(2^{k}-1\right)$ winning the DA-CMA game when issuing $q_{H_{i}}$ queries to *H*_*i*_ (*i* = 1, 2, 3), *q*_*ke*_ key extraction queries, *q*_*gsk*_ generation symmetric key queries, *q*_*ke*_ key encapsulation queries, and *q*_*kd*_ key decapsulation queries in a time *t*, C resolves BDH problem in expected time $t\leq 120686q_{H_{3}}q_{H_{1}}2^{k}/\epsilon_{datk}\left(2^{k}-1\right)$.

*Proof*: Refer to Appendix 4.

## 7 Performance

We conduct a main computational cost comparison of the construction with existing schemes LZJ [35] and HDA-I of LHO [39] listed in Table 1. The point multiplication in *G*_1_, the exponentiation calculation in *G*_2_, the addition calculations in *G*_1_, and the pairing calculation in *G*_2_ are denoted by PM, EC, AD, and PC, respectively. We ignore XOR, and hash function since they are trivial. In all computational cost, the PC evaluation is the most time-consuming. From Table 1, it shows that the computation overhead of our scheme is less than that of LZJ [35], but more than that of the HDA-I of LHO [39]. It is noted that LZJ [35] is not a heterogeneous DAE scheme which is not catered for the LBS and HDA-I of LHO [39] cannot achieve confidentiality.

**Table 1:** Performance comparison.

Schemes	Computational cost				Security		Heterogeneity
	PM	BP	AD	EP	DA-CMA	IND-CCA2	
LZJ [35]	4	3	1	1	√	√	×
HDA-I of LHO [39]	3	2	1	0	×	√	√
Ours	3	3	0	1	√	√	√

An experiment is conducted on the PBC library with A pairing [49]. The A pairing is designed on an elliptic curve *y*^2^ = *x*^3^ + *x* mod *p* for some prime *p* ≡ 3 mod 4. As needed, we set the order of *G*_1_ is *q* and the library’s embedding degree to 2. Here, 80-bit, 112-bit, and 128-bit denotes three kinds of AES [50] key size security level, respectively. Table 2 shows the description for different security levels.

**Table 2:** Description for different security level.

Security level	Size of *P*	Size of *q*
80-bit	512	160
112-bit	1024	224
128-bit	1536	256

**Fig 4:**
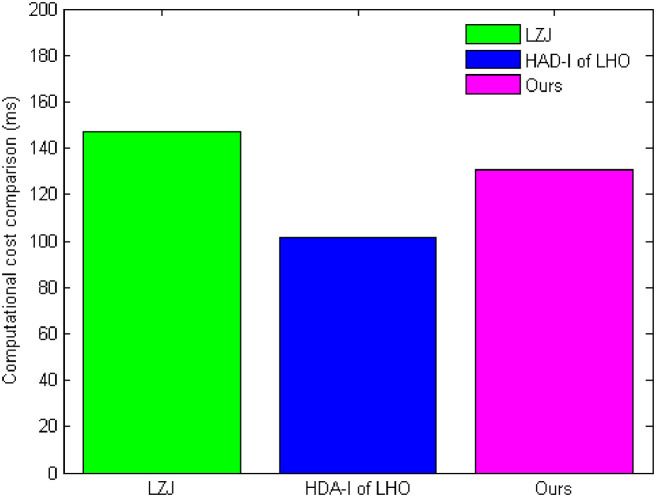
Computational cost comparison.

We implement the experiment on an Intel Pentium(R) with 2,048 MB of RAM (2,007.04 MB available) and Dual-Core processor running at 2.69 GHz. On this machine, a PM takes 15.927 ms, and an AD requires 0.065ms employing an ECC with *q* of 160 bits. A PC and an EC take 26.68 ms and 3.126 ms, respectively. LZJ [35] takes 146.939 ms, HDA-I of LHO [39] takes 101.206 ms, and our scheme takes 130.947 ms. Fig 4 depicts the comparative computational cost for LZJ [35], HDA-I of LHO [39], and our scheme. From Fig 4, we can see that the implementation results are consistent with the theoretical analysis.

**Fig 5:**
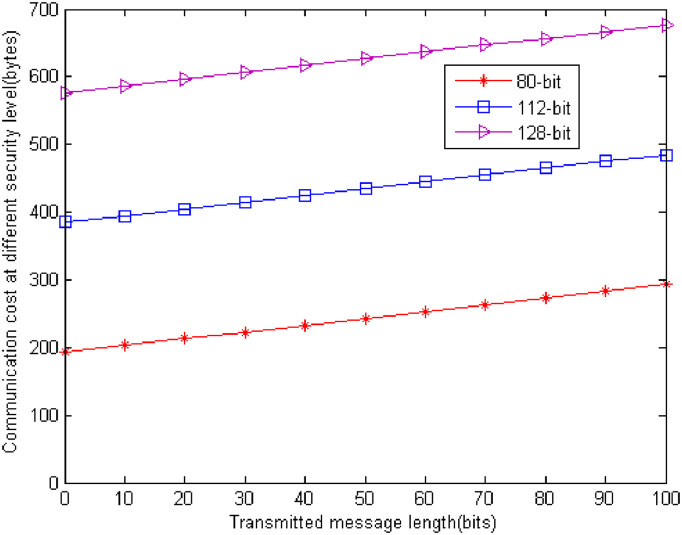
Communication cost at different security level.

For the communication cost, LZJ [35], HDA-I of LHO [39], and our scheme are |*m*| + |*G*_1_| + |*G*_2_|. They possess the identical communication cost. |*x*| is the size of *x*. For 80-bit security level, |*p*| = 512bits, |*G*_1_| = 1024bits, |*q*| = 160bits. If the standard compression techniques are used, *G*_1_ can be reduced to 65bytes. *G*_2_ = 1024bits = 128bytes. Therefore, the communication cost of the three schemes is |*m*| + |*G*_1_| + |*G*_2_| = | *m*| + 65 + 128 = |*m*| + 193bytes. For 112-bit security level, |*p*| = 1024bits, |*G*_1_| = 2048bits, |*q*| = 224bits. Using the standard compression technique, *G*_1_ can be reduced to 129bytes. *G*_2_ = 2048bits = 256bytes. Therefore, the communication cost of the three schemes is |*m*| + |*G*_1_| + |*G*_2_| = |*m*| + 129 + 256 = |*m*| + 385bytes. For 128-bit security level, |*p*| = 1536bits, |*G*_1_| = 3072bits, |*q*| = 256bits. Using the standard compression technique, *G*_1_ can be reduced to 193bytes. *G*_2_ = 3072bits = 384bytes. Therefore, the communication cost of the three schemes is |*m*| + |*G*_1_| + |*G*_2_| = |*m*| + 193 + 384 = |*m*| + 577bytes. Fig 4 shows the communication cost at different security level. It shows that from Fig 5 the 80-bit security level is our best choice for the current computing condition.

## 8 Application

Zeng et al. [51] presented a deniable ring authentication for protecting the LBS privacy. In their scheme, the user’s identity is anonymous to the LBSP and he/she can deny that he/she sends the requested location information to LBSP. However, the entities are all in the same environment and the requested location information is sent in plaintext. Any adversary can monitor or intercept this sensitive information. Therefore, to better resolve this issue, utilize our designed HDAE scheme in LBS systems to render the transmitted message in ciphertext. The specific communication process is as follows:

A user in a PKI environment wants to request the location-based service *m* from the service provider (SP) in an identity-based environment. It first executes the PKI-KG algorithm to produce its private/public key pair (*sk*_*s*_, *pk*_*s*_) and executes DAE(*m*, *sk*_*s*_, *pk*_*s*_, *ID*_*r*_) to create a ciphertext *σ*. The user then passes the resulting *σ* to the SP. When the SP receive the LBS request, it first requests a private key $S_{ID_{r}}$ from the PKG. Then it executes DAD($\sigma ,pk_{s},ID_{r},S_{ID_{r}}$) to get the LBS request *m*. It cannot send the response of *m* to any third party, since the third party cannot ensure whether the LBS request *m* is from the user or the service provider, due to the fact that the service provider can generate the same LBS request *m* and ciphertext *σ* with indistinguishable probabilities.

## 9 Conclusion

In this paper, we designed a hybrid DAE scheme which comprises a PI-HDAE scheme and a DEM scheme. The entities are in a heterogeneous system where the sender belongs to the PKI environment, while the receiver belongs to the IBC environment. Our construction can achieve confidentiality and deniable authentication in a single logic step. We give a formal security proof in the ROM. Our performance results show that this construction is secure and efficient. Furthermore, we present an example and apply our design to LBS system for better service.

**Appendix 1**

*Proof*: Our proof strategy is shown below. The modified games *Game*_0_, *Game*_1_, *Game*_2_ are defined in [52, 53]. The games’ difference lies in how the environment replies F’s queries. F receives the challenge ciphretext σ* = (ϕ*, c*) that encrypts either *m*_0_ or *m*_1_ by its challenge oracle in the light of *b* utilizing symmetric key K*. K* is also used in the decapsulation ϕ* with *pk*_*s*_ and *ID*_*r*_ chosen by F. In *Game*_*i*_ (*i* = 0, 1, 2), it is supposed that *S*_*i*_ is the event *δ*′ = *δ*. F’s challenge oracle outputs *δ* and F returns *δ*′. F’s random oracle and F’s oracle determines the probability.

The lemma from [54] is employed as follows.

**Lemma 1**. Let *E*, *E*′, and *F* be events defined on a probability space such that *Pr*[*E*∧¬*F*] = *Pr*[*E*′∧¬*F*]. Then, we get |*Pr*[*E*] − *Pr*[*E*′]| ≤ *Pr*[*F*].

*Game*_0_: We execute key extraction algorithm to simulate adversary’s view in a real attack. Then we utilize the produced key to reply F’s queries. Thus, the adversary’s view is identical to it in a real attack. Hence, we find

$$|Pr\left[S_{0}\right]-\frac{1}{2}|=\frac{1}{2}Adv_{PI-HDAE}^{IND-CCA2}\left(F\right).$$

*Game*_1_: In this game, we only alter how the DAD oracle replies F’s queries. After the calling of the challenge DAE oracle, (*ϕ*, *c*), *pk*_*s*_ and *ID*_*r*_ are submitted to the DAD oracle. If $pk_{s}=pk_{s}^{*}$, $ID_{r}=ID_{r}^{*}$, *ϕ* = ϕ*, the DAD oracle does not employ the key *K*, and it utilizes the key K* to decapsulate *c* and passes the result to F.

This change does not affect F and so

$$Pr\left[S_{1}\right]=Pr\left[S_{0}\right].$$

**Lemma 2**. The running time of a ppt algorithm $C_{1}$ is identical to that of F, so we have

$$|Pr\left[S_{2}\right]-Pr\left[S_{1}\right]|=Adv_{PI-HDATK}^{IND-CCA2}\left(C_{1}\right).$$

*Proof*: The proof below gives how to design $C_{1}$ of the PI-HDATK to be against the IND-CCA2 attack.

The game is between $C_{1}$ and F as follows.

- *Setup*: $C_{1}$ passes the *param* to F. Additionally, it also passes the sender’s public key *pk*_*s*_ to F.
- *Phase* 1: F submits a receiver’s identity *ID*_*j*_ to $C_{1}$. $C_{1}$ executes a key extraction (KE) query to its own oracle and transmits the response to F. When F executes an encryption query on *m*, and *ID*_*j*_, $C_{1}$ works as follows.
  1. Issue a symmetric key generation (SKG) query on *ID*_*j*_ to gain *K*.
  2. Calculate *c* = *DEM*.*Enc*(*K*, *m*).
  3. Issue a key encapsulation (KES) query on *c* to gain *ϕ*.
  4. Return *σ* = (*ϕ*, *c*).

When F executes a key decryption (KD) query on *σ* = (*ϕ*, *c*), and *ID*_*j*_, $C_{1}$ works as follows.

1. Issue a KD query on (*ϕ*, *c*, *ID*_*j*_) to get *K*.
2. If *K* = ⊥, abort.
3. Calculate *m* = *DEM*.*Dec*(*K*, *c*) and output *m*.

- *Challenge*: F produces a challenge identity *ID*_*j*_ and messages (*m*_0_, *m*_1_) with equal-lengths. $C_{1}$ works as follows.
  1. Pass *ID*_*j*_ to its challenger to gain *K*_*β*_ for *β* ∈ {0, 1}.
  2. Elect *δ* ∈ {0, 1}.
  3. Compute c* = *DEM*.*Enc*(*K*_*δ*_, *m*_*δ*_).
  4. Pass c* to its challenger to gain ϕ*.
  5. Return σ* = (ϕ*, c*) to F.
- *Phase* 2: F issues queries just like in phase 1 except for requesting a KE query on *ID*_*r*_ and a KD query on σ* = (ϕ*, c*) to gain the corresponding message.
- *Guess*: F returns *δ*′. If *δ*′ = *δ*, $C_{1}$ returns *b*′ = 1 which means *K*_*b*_ is a genuine key; or else it returns *b*′ = 0 which means *K*_*b*_ is a random key.

When *K*_*b*_ is a genuine key, F is performed just like it in *Game*_1_. It means

$$Pr\left[S_{1}\right]=Pr\left[\delta^{\prime }=\delta |b=1\right]=Pr\left[b^{\prime }=1|b=1\right].$$

When *K*_*b*_ is a random key, F is executed just like it in *Game*_2_. It implies

$$Pr\left[S_{1}\right]=Pr\left[\delta^{\prime }=\delta |b=0\right]=Pr\left[b^{\prime }=1|b=0\right].$$

Based on PI-HDATK’s security definition, we receive

$$Adv_{PI-HDATK}^{IND-CCA2}\left(C_{1}\right)=|2Pr[b^{\prime }=b]-1|=|Pr\left[b^{\prime }=1|b=1\right]-Pr\left[b^{\prime }=1|b=0\right]|.$$

**Lemma 3**. The running time of a ppt algorithm $C_{2}$ is identical to that of F, so

$$|Pr\left[S_{2}\right]-\frac{1}{2}|=\frac{1}{2}Adv_{DEM}^{IND-PA}\left(C_{2}\right).$$

*Proof*: The proof below gives how to design $C_{2}$ of the PI-HDATK to be against the IND-PA attack. F is run just like the manner in game *Game*_2_. Before F calls its challenge DAE query, we perform the key extraction algorithm to answer F’s query. When F issues its challenge DAE query on identity $ID_{r}^{*}$, and two messages (*m*_0_, *m*_1_), we just transfer (*m*_0_, *m*_1_) to $C_{2}$’s challenge encapsulation oracle to gain c*. We then issue a GSK query to have K* and issue a KES query to have ϕ*. We transmit (ϕ*, c*) to F and drop K*.

*Pr*[*S*_2_] is the probability that $C_{2}$ pinpoints the challenge encapsulation oracle’s hidden bits due to that $C_{2}$ returns whatever F returns.

**Appendix 2**

*Proof*: F attacks the PI-HDAE scheme with advantage $Adv_{PI-HDAE}^{DA-CMA}\left(F\right)$. C attacks DA-CMA for PI-HDATK with advantage at least $Adv_{PI-HDAE}^{DA-CMA}\left(F\right)$. We issue F’s queries below.

- *Setup*: C passes the *param* to F. Additionally, C also transmits *pk*_*s*_ to F.
- *Attack*: When F submits an *ID*_*j*_ to C, C executes a KE query to its own oracles and passes the response to F. When F performs a DAE query on *m*, and *ID*_*j*_, C issues the SKG query, KES query and KD query just like $C_{1}$ works in Lemma 2.
- *Fogery*: F outputs $\left(m^{*},\sigma^{*},ID_{r}^{*}\right)$, where σ* = (ϕ*, c*). C returns $\left(\tau^{*},\phi^{*},ID_{r}^{*}\right)$, where τ* = c*.

Visibly, this is a perfect proof. If F wins the DA-CMA game for PI-HDAE, C has the identical advantage to win the DA-CMA game for PI-HDATK.

**Appendix 3**

*Proof*: C gets an input (*P*, *aP*, *bP*, *cP*) of DBDH problem and purposes to decide if *θ* = *e*(*P*, *P*)^*abc*^. C is a challenger and performs F as a subroutine. C responds to F’s queries on *H*_1_, *H*_2_ and *H*_3_ and these answers are created randomly. C reserves lists *L*_1_, *L*_2_ and *L*_3_ to keep the answers. The assumptions are made as follows.

1. Before F issues KE queries, GSK queries, KES queries and KD queries on identity *ID*, F will first inquire *H*_*ID*_.
2. A KES query’s encapsulation ciphertext will not be employed in a KD query.

- *Setup*: C transmits system parameters with *P*_*pub*_ = *cP* to F in which *c* is unknown to C. Additionally, C produces sender’s (*sk*_*s*_, *pk*_*s*_) and transmits public key *pk*_*s*_ to F.
- *Phase 1*: F issues queries as follows.
  - *H*_1_ *queries*: C picks $\gamma \in \{1,2,\ldots ,q_{H_{1}}\}$. F requests *H*_1_ queries on its choice identities. At the *γ*-th query, C replies by *H*_1_(*ID*_*γ*_) = *bP*. At the *j*-th query with *j* ≠ *γ*, C picks $w_{j}\in Z_{q}^{*}$, adds (*ID*_*j*_, *w*_*j*_) in the list *L*_1_ and responds *H*_1_(*ID*_*j*_) = *w*_*j*_ *P*.
  - *H*_2_, *H*_3_ *queries*: When F issues hash value queries, C checks whether the corresponding items are included in the lists. If yes, F will get the same answer; otherwise, F will get a random value. The value and query will be added in the list.
  - *Key extraction queries*: When F issues key extraction queries on receiver’s identity *ID*_*j*_. If *ID*_*j*_ = *ID*_*γ*_, C aborts. If not, *L*_1_ must comprise (*ID*_*j*_, *w*_*j*_) (it implies C has replied *H*_1_(*ID*_*j*_) = *w*_*j*_ *P*.) The private key *cH*_1_(*ID*_*j*_) = *w*_*j*_ *cP* = *w*_*j*_ *P*_*pub*_ is calculated by C and transmitted to F.
  - *Generation symmetric key queries*: F submits an *ID*_*j*_ to C. C then executes (*K*, *ω*) = *Sym*(*sk*_*s*_, *pk*_*s*_, *ID*_*j*_) and passes *K* to F. C saves *ω* and overwrites the previous value.
  - *Key encapsulation queries*: F creates *τ*. C checks if *ω* already exists. If not, C aborts. Or else, C just executes *ϕ* = *Encap*(*ω*, *τ*) and transmits the encapsulation ciphertext *ϕ* to F.
  - *Key decapsulation queries*: F sends the receiver’s identity *ID*_*j*_, a tag *τ*, and an encapsulation *ϕ*. If *ID*_*j*_ = *ID*_*γ*_, (*ϕ*, *τ*) is invalid. If F requests *H*_3_(*t*, *τ*, *pk*_*s*_, *ID*_*j*_), where $t=W/e(V,S_{ID_{j}})$, C replies *h* that coincides with *V* = *hpk*_*s*_, it aborts. From F’s perspective, *σ* = (*W*, *V*) is valid. The probability is at most 1/2^*k*^. If *ID*_*j*_ ≠ *ID*_*γ*_, C gains $S_{ID_{j}}$ by performing the key extraction query. It then passes the result of $Decap(\sigma ,\tau ,S_{ID_{j}})$ to F.
- *Challenge*: F determines when phase 1 is over. It generates a receiver’s challenge identity *ID*_*r*_. If F has issued a key extraction query on *ID*_*γ*_, C aborts. If F does not pick *ID*_*r*_ = *ID*_*γ*_ as the target identity, it aborts too. C picks W* ∈ *G*_2_, sets V* = *aP* and computes t* = W*/*θ* (*θ* is DBDH problem’s candidate). Then C issues *H*_2_ query to look for *K*_1_ = *H*_2_(t*). C randomly picks *K*_0_, *β* ∈ (0, 1), and passes *K*_*β*_ to F. F then passes τ* to C. Whereafter, C transmits σ* = (W*, V*) to F.
- *Phase 2*: F issues queries as in phase 1 except that it has no ability to issue a KE query on *ID*_*r*_ and a KD query on (ϕ*, τ*) to gain the symmetric key.
- *Guess*: F outputs *β*′ for (*K*_*β*_, ω*) = *Sym*(*sk*_*s*_, *pk*_*s*_, *ID*_*r*_) and ϕ* = *Encap*(ω*, τ*) hold. If *β*′ = *β*, C outputs 1 shows *θ* = *e*(*P*, *P*)^*abc*^; If not, C outputs 0 shows *θ* ≠ *e*(*P*, *P*)^*abc*^.

Now we calculate C’s successful probability. If one of the events below is satisfied, C will fail:

- *E*_1_ F does not pick *ID*_*γ*_ as the receiver’s identity in challenge phase.
- *E*_2_ F has issued a KE query on *ID*_*γ*_.
- *E*_3_ C terminates in a KD query due to it refuses a valid encapsulation.

We show that Pr[¬*E*_1_] = 1/$q_{H_{1}}$, and Pr[*E*_3_] ≤ *q*_*kd*_/2^*k*^. Additionally, ¬*E*_1_ means ¬*E*_2_.

Because

*p*_1_ = Pr[*β*′ = *β*∣(*K*_*β*_, ω*) = Sym(*pk*_*s*_, *sk*_*s*_, *ID*_*r*_)] and $\phi *=Encap(\omega *,\tau *)=\frac{\epsilon +1}{2}-\frac{q_{kd}}{2^{k}}$

and

$p_{0}=\text{Pr}[\beta^{\prime }=i|\theta \in_{R}G_{2}]=\frac{1}{2}$ for *i* = 0, 1,

We get

$$\text{Adv}(C)=\frac{\left|p_{1}-p_{0}\right|}{q_{H_{1}}}=(\frac{\epsilon +1}{2}-\frac{q_{kd}}{2^{k}}-\frac{1}{2})(\frac{1}{q_{H_{1}}})=\frac{\epsilon -q_{kd}/2^{k-1}}{2q_{H_{1}}}$$

*O*(*q*_*gsk*_ + *q*_*ke*_ + *q*_*kd*_) is C’s computation time that shows pairing computations in GSK queries, KE queries and KD queries.

**Appendix 4**

*Proof*: we have to let our design fit into the signature scheme described in [54], where the simulation step can be simulated in the absence of the sender’s private key (i.e., absence of the master private key). On this occasion, we need an approach to resolve the BDH problem.

First, we observe that the PI-HDATK scheme accords with the requested three-phase honest-verifier zero-knowledge identification protocol, where *σ*_1_ = *t* is the commitment, *h* = *H*_3_(*τ*, *t*, *pk*_*s*_, *ID*_*r*_) is the hash value, and *σ*_2_ = *W* is the answer.

Second, a simulation step is shown and an approach of how to resolve the BDH problem is given. Given (*P*, *aP*, *bP*, *cP*) of BDH problem, C needs to compute *h* = *e*(*P*, *P*)^*abc*^. C performs F as a subroutine. F consults C to reply *H*_1_, *H*_2_, and *H*_3_ and C holds *L*_1_, *L*_2_, and *L*_3_ to preserve the resulting responses. The process below is depicted.

- *Setup*: C calculates params with *P*_*pub*_ = *cP* and passes them to F. Additionally, C also transmits *pk*_*s*_ = *aP* to F.
- *Attack*: F executes the following queries.
  - *H*_1_ *queries* C picks $\gamma \in \{1,2,\ldots ,q_{H_{1}}\}$. F requests *H*_1_ queries on its choice identities. At the *γ*-th query, C replies by *H*_1_(*ID*_*γ*_) = *bP*. At the *j*-th query with *j* ≠ *γ*, C picks $w_{j}\in Z_{q}^{*}$, inserts (*ID*_*j*_, *w*_*j*_) in the list *L*_1_ and responds *H*_1_(*ID*_*j*_) = *w*_*j*_ *P*.
  - *H*_2_, *H*_3_ queries, KE queries, GSK queries, KES queries, and KD queries are identical to them in Theorem 3.
- *Fogery*: F outputs a triple (σ*, τ*, *ID*_*γ*_), where σ* = (W*, V*). We coalesce *ID*_*γ*_ and τ* into a “generalized” forged tag (*ID*_*γ*_, τ*) to hide the identity-based aspect of the DA-CMA attack, and simulate the setting of an identity-less adaptive-CMA existential forgery. If F is an efficient forger, then we have the capability to constitute a Las Vegas machine $F^{\prime }$ that outputs ((*ID*_*γ*_, τ*), h*, σ*) and $\left(\left(ID_{\gamma },\tau^{*}\right),{\bar{h}}^{*},{\bar{\sigma }}^{*}\right)$ with $h^{*}\neq {\bar{h}}^{*}$ and the same commitment t*. To resolve the BDH problem based on the machine $F^{\prime }$, we constitute a machine $C^{\prime }$ as follows.

1. $C^{\prime }$ performs $F^{\prime }$ to gain two distinct signatures ((*ID*_*γ*_, τ*), h*, σ*) and ($\left(ID_{\gamma },\tau^{*}\right),{\bar{h}}^{*},{\bar{\sigma }}^{*}$).
2. $C^{\prime }$ computes *e*(*P*, *P*)^*abc*^ as ${\left(W^{*}/{\bar{W}}^{*}\right)}^{1/(h^{*}-{\bar{h}}^{*})}$.

From the forking lemma [54] and the lemma on relationship between given-identity and chosen-identity attack [55], if F succeeds with probability $\epsilon_{datk}\geq 10\left(q_{ke}+1\right)\left(q_{ke}+q_{H_{3}}\right)q_{H_{1}}/\left(2^{k}-1\right))$ in time *t*, then $C^{\prime }$ resolves the BDH problem in expected time $t\leq 120686q_{H_{3}}q_{H_{1}}2^{k}/\epsilon_{datk}\left(2^{k}-1\right)$.

## Supporting information

S1 File (DOCX)
