# Supplementary material for: Heterogeneous deniable authenticated encryption for location-based services
Source: PLoS One. 2021 Jan 6;16(1):e0244978. doi: 10.1371/journal.pone.0244978 (PMC7787476; doi:10.1371/journal.pone.0244978)
Supplement: S1 File — (DOCX) [file pone.0244978.s001.docx]

We conduct a main computational cost comparison of the construction with existing schemes LZJ [32] and HDA-I of LHO [36] listed in Table 1. The point multiplication in G1, the exponentiation calculation in G2, the addition calculations in G1, and the pairing calculation in G2 are denoted by PM, EC, AD, and PC, respectively. We ignore XOR, and hash function since they are trivial. In all computational cost, the PC evaluation is the most time-consuming. From Table 1, it shows that the computation overhead of our scheme is less than that of LZJ [32], but more than that of the HDA-I of LHO [36]. It is noted that LZJ [32] is not a heterogeneous DAE scheme which is not catered for LBS and HDA-I of LHO [36] cannot achieve confidentiality.

An experiment is conducted on the PBC library with A pairing [43]. The A pairing is designed on an elliptic curve y2 = x3 + x mod p for some prime p$\equiv$3 mod 4. As needed, we set the order of G1 is q and the library's embedding degree to 2. Here, 80-bit, 112-bit, and 128-bit denotes three kinds of AES [44] key size security level, respectively. Table 2 shows the description for different security level.

We implement the experiment on an Intel Pentium(R) with 2,048 MB of RAM (2,007.04 MB available) and Dual-Core processor running at 2.69 GHz. On this machine, a PM takes 15.927 ms, and an AD requires 0.065ms employing an ECC with q of 160 bits. A PC and an EC take 26.68 ms and 3.126 ms, respectively. LZJ [32] takes 146.939 ms, HDA-I of LHO [36] takes 101.206 ms, and our scheme takes 130.947 ms. Fig. 4 depicts the comparison computational cost for LZJ [32], HDA-I of LHO [36], and our scheme. From fig. 4, we can see that the implementation results are consistent with the theoretical analysis. For the communication cost, LZJ [32], HDA-I of LHO [36], and our scheme are |m|+|G1|+|G2|. They possess the identical communication cost. |x| is the size of x. For 80-bit security level, |p|=512bits, |G1|=1024bits, |q|=160bits. If the standard compression techniques are used, G1 can be reduced to 65bytes. G2=1024bits=128bytes. Therefore, the communication cost of three scheme is |m|+|G1|+|G2|=|m|+65+128=|m|+193bytes. For 112-bit security level, |p|=1024bits,|G1|=2048bits, |q|=224bits. Using the standard compression technique, G1 can be reduced to 129bytes. G2=2048bits=256bytes. Therefore, the communication cost of three scheme is |m|+|G1|+|G2|=|m|+129+256=|m|+385bytes. For 128-bit security level, |p|=1536bits, |G1|=3072bits, |q|=256bits. Using the standard compression technique, G1 can be reduced to 193bytes. G2=3072bits=384bytes. Therefore, the communication cost of three scheme is |m|+|G1|+|G2|=|m|+193+384=|m|+577bytes. Fig. 4 shows the communication cost at different security level. It shows that from Fig. 5 the 80-bit security level is our best choice for the current computing condition.
